# Harnessing Machine Learning to Personalize Web-Based Health Care Content

**DOI:** 10.2196/25497

**Published:** 2021-10-19

**Authors:** Ahmad Guni, Pasha Normahani, Alun Davies, Usman Jaffer

**Affiliations:** 1 Department of Surgery and Cancer Imperial College London London United Kingdom; 2 Imperial Vascular Unit Imperial College Healthcare NHS Trust London United Kingdom

**Keywords:** internet, online health information, personalized content, patient education, machine learning

## Abstract

Web-based health care content has emerged as a primary source for patients to access health information without direct guidance from health care providers. The benefit of this approach is dependent on the ability of patients to access engaging high-quality information, but significant variability in the quality of web-based information often forces patients to navigate large quantities of inaccurate, incomplete, irrelevant, or inaccessible content. Personalization positions the patient at the center of health care models by considering their needs, preferences, goals, and values. However, the traditional methods used thus far in health care to determine the factors of high-quality content for a particular user are insufficient. Machine learning (ML) uses algorithms to process and uncover patterns within large volumes of data to develop predictive models that automatically improve over time. The health care sector has lagged behind other industries in implementing ML to analyze user and content features, which can automate personalized content recommendations on a mass scale. With the advent of *big data* in health care, which builds comprehensive patient profiles drawn from several disparate sources, ML can be used to integrate structured and unstructured data from users and content to deliver content that is predicted to be effective and engaging for patients. This enables patients to engage in their health and support education, self-management, and positive behavior change as well as to enhance clinical outcomes.

## Introduction

The internet is a key medium in the consumption of health care–related content. Two-thirds of internet users in the United Kingdom and the United States access health-related information on the internet [1-3]. Furthermore, patients are increasingly motivated and able to participate in developing this growing repository of information by sharing their lived experiences [4]. Health care professionals also consume as well as create and share web-based health care information [5].

A vast array of web-based content types and delivery media and channels are available, including videos, webpages, podcasts, images, online discussion groups and communities, and social media [6-10]. A systematic review reporting on the assessment of web-based content quality identified key domains, including accuracy, completeness, accessibility, presentation, and design, which were important overall in determining how useful and engaging content was for patients [11]. However, there is considerable variability in the quality of such content [12]. Berland et al [13] demonstrated that using search engines for common health conditions retrieved relevant content in only one out of five searches, suggesting that patients are likely to come across irrelevant content when seeking information about health. Moreover, only half of the topics that physicians thought important to convey were accurately and appropriately covered [13].

The concept of content personalization is a powerful approach that addresses the previously described features of quality by presenting the user with relevant information that is both appropriate and engaging. A more engaged patient is more likely to understand information about their health, partake in healthy behaviors, and adhere to treatment, leading to better health outcomes [14].

Machine learning (ML) is a subset of artificial intelligence that uses algorithms to study patterns in data and develop models that improve predictions about the data over time through supervised learning, unsupervised learning, or reinforcement learning [15]. Many industries use ML techniques to analyze accrued *big data* to personalize content for users [16]. The health care sector may be well served by considering these advances in other industries to personalize experiences for people seeking health care content. ML-assisted personalization can be considered for both large groups and populations or for individuals.

In this review, we aim to first outline why the health care sector should recognize the importance of personalizing content (*Why Personalizing Web-Based Health Care Content Is Important*). We then explore the current landscape of content personalization (including ML and non-ML) both within and outside health care (*Content Personalization*). Finally, we discuss practical applications of personalization in health care, outline a model that demonstrates how ML can personalize web-based content, and consider the anticipated benefits and drawbacks (*Potential Lessons to Learn for Health Care*).

## Why Personalizing Web-Based Health Care Content Is Important

There has been an increased focus on empowering patients to engage with their own health. The delivery of information to patients has been recognized as a tenet of health care policy, resulting in almost universally positive outcomes for patients, health care staff, and communities [17]. The UK National Health Service Five Year Forward View outlined the need to facilitate patient activation by improving access to information, supporting self-management, and increasing patient control over the care they receive, with particular emphasis placed on harnessing digital technology [18]. This aligns with the *patient-centered* model [19], which improves patient satisfaction, quality of life, and quality of care provided [20]. Personalization facilitates the patient-centered model by delivering health care content that accounts for the preferences and needs of individual patients. The proliferation of easily accessible web-based content provides an opportunity to enable *patient-centered* information delivery at scale.

A randomized controlled trial of the provision of computer-based information to cancer patients reported that patients preferred to receive personalized information (based on their medical records) as opposed to generalized information [21]. They were more likely to share these resources with family members, and additionally, this approach was associated with a reduction in anxiety levels. A similar effect was demonstrated with personalization of booklets [22] and tailored information packs [23].

It is well established that the health care content needs to vary between different patients and also change over time. Uncertainty, the inability to determine the meaning of illness-related events, has been shown to have a deleterious effect on patient experience and outcomes [24,25]; therefore, timely and accurate delivery of information is important to address information needs. However, patients’ information needs vary according to stage of disease, stage of patient journey, age, previous experiences, and coping styles [26]. A blanket *one-size-fits-all* strategy for designing and delivering health care content is unlikely to be effective.

Another advantage of personalizing health care content is its potential to improve health-related choices. One of the principles of the *patient-centered* model is sharing responsibility for clinical decisions with patients (shared decision-making) [27]. Patient decision aids are evidence-based tools designed to assist in shared decision-making. They facilitate information exchange by helping patients understand the clinical conditions and the available options for treatment. They have been demonstrated to improve patient knowledge and facilitate decision-making that is more aligned with patient values and preferences [28,29].

A study on improving patient decision-making related to prostate cancer screening found that personalizing a patient decision aid based on a number of factors that patients considered important (eg, survival, unnecessary biopsy, overdiagnosis, quality of life, burden of treatment, and burden on caregivers) improved patient opinion on screening and the quality of their decision [30]. Decision quality was assessed using an instrument that allows patients to self-rate and weigh separate elements of decision quality, including the perceived clarity of options provided, relative importance and likelihood of possible outcomes, trust toward the information delivered, support received throughout the decision-making process, sense of control over the decision, and commitment toward acting on the decision [31].

It is increasingly recognized that delivering health information without consideration for personalization and the relevance of content experienced limits the potential to change health behavior [32,33]. A meta-analysis on behavior choices from 40 web-based interventions, which used personalized strategies including interactive multimedia content, tailored feedback, discussion groups, and personalized management plans, showed a positive impact on behavior outcomes related to smoking cessation, alcoholism, physical activity, diet, and chronic disease management [34]. These findings are corroborated by other meta-analyses evaluating tailored content for similar health-related behavior outcomes [35-38]. However, given the significant heterogeneity in the intervention modality, design, and features, it is challenging to identify the specific factors that are most associated with behavior change.

With a greater understanding of these factors, there is significant scope to integrate personalized content into both large-scale public health initiatives as well as individual treatment plans to encourage self-management, adherence to treatment, and positive lifestyle changes.

## Content Personalization

### Content Personalization in Health Care—Current State

The paths patients take to encounter web-based content can be described by a number of discrete patient journeys. First, patients can independently find web-based information using internet search engines. Although this offers patients a plethora of information, quality (as previously discussed in the *Introduction* section) is variable [12]. Without strict content moderation and regulation, patients may struggle to parse out factual and relevant content, instead relying on content that is superficially engaging (*clickbait*) or appears credible. Furthermore, subtle differences in search terms can significantly alter the quality of the retrieved information [39].

Health care organizations and services hold repositories of quality-controlled content and can serve as gateway sites for other similar websites [40]. These provide credible and accurate information but hold limited quantities of content and may not be directly relevant to every patient. Health care professionals can assess individual information-seeking needs during consultations and refer patients to high-quality and engaging content [5]. However, this solution lacks scalability because most web-based health care searching encounters are *unsupervised* by health care providers. Limitations on how patients access health information can be addressed with content personalization, which mandates an understanding of what factors may be important in personalizing content.

Patients’ information needs are affected by several factors that may influence how patients respond to web-based content, as discussed in *Why Personalizing Web-Based Health Care Content Is Important* section. For example, in the context of age, older patients often report difficulty in accessing useful web-based content because of complex website layouts, lack of navigational aids or instructional tools, and too much information being presented [41]. Younger patients may be more prone to uncertainty and worry about their health, resulting in information-seeking behavior [42]. A study that allowed cancer patients to self-tailor web-based educational content based on text, visual, and audio-visual modes demonstrated increased satisfaction among younger patients in comparison with nontailored content [43].

With regard to factors that affect the decision to select or reject web-based content, a study found several content and design features that influenced whether patients trust web-based information related to hormone replacement therapy [44]. An initial poor impression of design factors—including inappropriate website name, complex layout, poor navigation aids, dull design, small print, and excessive text—constituted 94% of cited reasons for rejection. Content features were then comparatively more important in selecting trustworthy websites. This consisted of informative content, accessible explanations, illustrations, breadth of topics covered, unbiased information, age-related information, clear language, discussion groups, and a frequently answered questions section. Source factors were also key, such as explicit author or organization credibility and authors with similar social identities.

Other studies have evaluated the design and content factors that influence patients’ engagement in web-based videos, particularly on the video streaming website YouTube, which is one of the most popular websites with over 2 billion daily views [45]. These include educational resources on a range of medical topics for both patients and health care professionals [46-51]. These studies also assessed the quality of content uploaded on YouTube, which is not strictly regulated and is liable to misinformation [7]. However, the correlation between engagement and quality of content is conflicting [7], suggesting that other factors are important for gaining user attention in educational resources.

An analysis of 390 scientific communication videos on YouTube found that user-generated content, videos with regular presenters, and rapidly paced videos were more engaging than their counterparts [52].

Similarly, another study concluded that patient experience videos were more popular than videos created by health care professionals, as assessed by the video power index [53]. The video power index is an innovative tool that measures video performance by assessing its effectiveness on all platforms, comparing it with industry leaders, and aiding strategies to engage target audiences [54]. In terms of webpage content, Finnegan et al [55] found that engaging content categories were first-person narrative articles, articles that answer questions posed by readers, and articles with videos embedded in the webpage. These are all potential factors that can be considered when personalizing video content toward patients.

Sorice et al [56] examined patients’ preferred social media content related to plastic surgery on six social media platforms (Facebook, Instagram, Pinterest, Snapchat, Twitter, and YouTube) [56]. Patients used Facebook and YouTube as the most favored posts relating to before and after photographs and the surgery practice information. Second, the content that engaged plastic surgeons and patients differed. The authors concluded that this information should guide the web-based activity of plastic surgeons to effectively target the desired patients.

A systematic review evaluating factors associated with engaging web-based content revealed the following key categories: textual information, discussion boards and web-based groups, video content, visual or pictographs, device accessibility, stage of patient journey, credibility, and completeness of information [57]. A framework was developed for each category describing the factors that should be considered when designing an effective content. Evidently, the manner in which users engage with health care content is influenced by both design and content factors, many of which are likely not yet identified.

### Content Personalization Outside Health Care—Current State

With increasing volumes of web-based data available for extraction, storage, and processing, ML is useful in improving the efficiency and accuracy of data processing models without human input. Its application spans a wide range of disciplines, including marketing, engineering, computer science, finance, bioinformatics, and health care. In the context of personalizing health care content, ML applications may fall into the following categories: facilitating market segmentation, content analysis, and recommender systems.

In marketing, maximizing user—or customer—engagement is obviously a key driver. Customer segmentation and personalization of content in these segments in a competitive environment is easy to appreciate. Furthermore, 59% of customers believe that personalization influences their purchasing habits [58]. A study reporting over 30,000 campaigns by one company revealed that targeted campaigns resulted in greater customer retention, engagement, and conversion into active users compared with generic campaigns [59]. Audience segmentation for web-based marketing aims to split the customer population based on characteristic features (eg, demographic, psychographic, geographic, behavior, and product preference) [60]. Individual customer segments can be targeted with specific content and products predicted to elicit the most attention, resulting in sales and profits [61]. However, customer segmentation performed by human marketers is limited by the amount of data that can be amassed, analytical methods that can be used, and the number of conclusions drawn. ML using clustering techniques can process larger volumes of data and uncover complex patterns to draw more practical conclusions and create better-defined segments for targeting. Infamously, this approach can also be used to target groups with messages that may affect behavior, such as political elections [62], but is less likely to be a useful method to personalize health care content for individuals, as there will still be differences in the needs and preferences of individuals within segments.

Recommender systems are used by the entertainment, e-commerce, and marketing industry to personalize content discovery and information retrieval in the context of massive item repositories [63-66]. Established methods include collaborative filtering, which applies the behavior of similar users to suggest new items of interest; content-based methods, which analyze content similarities with previous user preferences to produce recommendations; and hybrid methods, which combine both. Although the research landscape has predominantly focused on collaborative filtering [67], increased interest has gathered around content-based filtering with techniques emerging to identify content features [68], including user-generated tags and reviews [69], and advances in video [70] and image [71] analysis capabilities.

As one of the largest platforms for creating and sharing content, the YouTube recommender system uses deep learning to generate and rank candidate videos by incorporating a rich set of user and video features, such as the user’s history, context, and interaction with similar videos [72]. This facilitates access to a small set of engaging personalized content from an ever-increasing repository of videos. Other studies have demonstrated several content factors that can also influence personalization. For example, a study incorporated textual content features including video metadata and nontextual features consisting of audio, scenes, and motion to enhance personalized recommendations for videos; this was more accurate in effective personalized video recommendation from large video data sets (Netflix and MovieLens) over existing models that use single specific content features [73].

Social media recommender systems provide insights into how companies personalize other media content discovery for users. Instagram analyzes content that users have previously interacted with and uses natural language processing to identify similar accounts to recommend content that the user is likely to interact with on their *Explore* page [74]. In addition, content analysis of social media pages reveals several factors that also influence user engagement and may further refine content personalization. In a study on over 13,000 Instagram posts, using an image application programming interface (API) to extract visual features from posts, several creator-related, context, and content factors predicted user engagement [75]. In particular, images containing people, scenery, and emoticons associated with positive emotions engaged users more strongly. Other content features on Instagram that correlate with user attention are photos with faces [76] and filters enhancing warmth, exposure, and contrast [77]. An analysis using a natural language processing API on over 100,000 messages on Facebook found that emotional and philanthropic content enhances engagement, whereas informative content reduces engagement in isolation, but further invokes attention when combined with persuasive features [78].

Advances in recommender systems have further improved the personalized recommendations. For instance, movie recommender systems traditionally use higher semantic features (eg, tags, plot, genre, and actors) suggested by users or experts to personalize recommendations [79]. A recent work using a deep learning neural network found that extracting low-level stylistic features (eg, colors, texture, and lighting) outperformed traditional semantic-based methods in recommending content [70]. With developments in algorithmic approaches and deep learning [68], high- and low-level content features can be integrated to generate more personalized content recommendations.

Recently, open-source services that leverage ML have become available on commercial platforms with the Google Cloud Artificial Intelligence as a foremost example [80,81]. These services require minimal ML expertise and consist of custom models using AutoML and pretrained models, which include video intelligence API (analyze video metadata), natural language API (analyze text), vision API (image segmentation and classification), and speech API (transcribing audio). Similar platforms exist with Amazon Rekognition image and video analysis [82], Microsoft Azure video indexer, text analytics and personalizer [83], and IBM Watson video content analysis and natural language understanding [84]. Amazon’s predictive user engagement service offers to improve user engagement by analyzing real-time activity to personalize recommendations and notifications for users [85,86]. The prospect of designing custom ML may have been prohibitive for many industries previously, but these open-source platforms provide an opportunity to adopt it into the mainstream of a variety of disciplines for large-scale data processing.

## Potential Lessons to Learn for Health Care

The previous sections described user segmentation, targeted advertisements, and personalization based on recommender systems using ML techniques. With the vast amount of web-based health care content readily accessible to patients, cross-disciplinary collaboration and the use of open-source platforms indicate that these techniques may be feasible. If this is achieved, the aim of personalizing web-based content and enhancing outcomes is possible. However, clinical studies and clinical applications related to this are sparse.

Big data in health care can transform the field of health marketing (an established concept in public health medicine), drawing principles from traditional marketing to create, communicate, and deliver information in a patient-centered manner [87]. This aims to identify population segments and market health care messages to them in terms of the segments that are likely to respond [88]. A systematic review of health marketing research identified a number of studies that used hierarchical and nonhierarchical clustering techniques to segment health consumers in unique ways [88]. However, the studies did not explore whether these segments were meaningful (predictive segmentation) or whether personalized interventions affected outcomes. Furthermore, there was a reliance on rudimentary data such as survey, service, and basic clinical data, which limits the clustering process as opposed to truly *big* data. Although these strategies may have beneficial effects for groups of people, it is difficult to imagine their utility to individuals.

**Figure 1:**
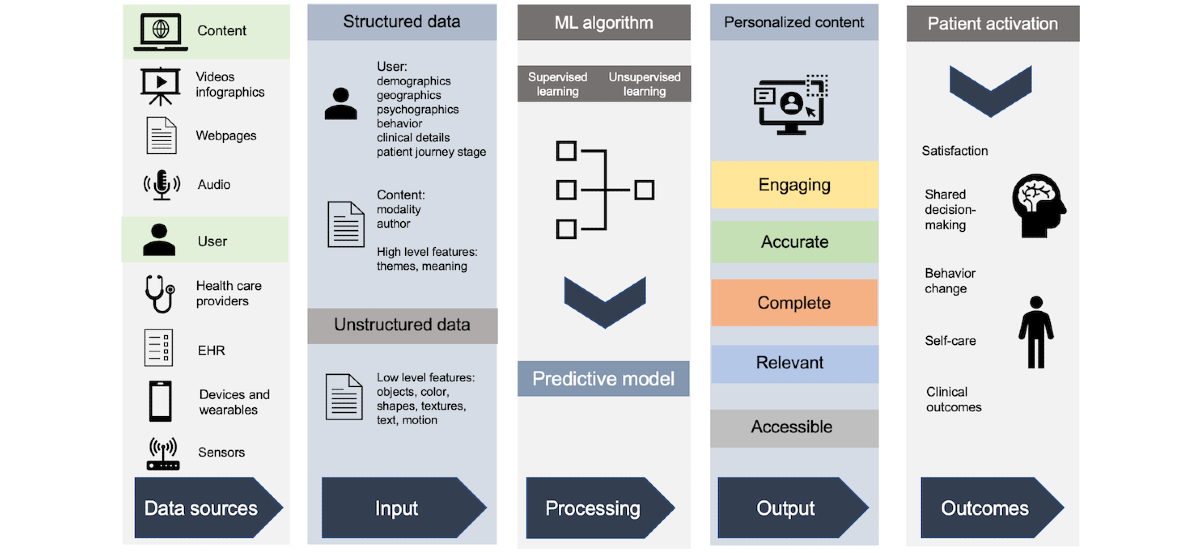
Suggested model using machine learning to personalize web-based health care content. EHR: electronic health record; ML: machine learning.

We propose a model that leverages ML algorithms to personalize content for an individual person (Figure 1). Health care big data consists of diverse data types, including clinical data, electronic patient records, biometrics, sensor-generated data, population data, social media posts, and webpages [16]. Electronic health records are accumulating data at an exponential rate. With the increasing use of medical devices, sensors, wearable technology, and social media, more personal data can be recorded [89]. These consist of potential sources of structured and unstructured data that may be fed into ML algorithms. Structured data include labeled user features such as demographics, geographics, psychographics, behavior, and clinical details, as well as content features consisting of modality, themes, and author information. Unstructured data, comprising 80% of all health care data [90], can be processed by video, image, and natural language processing APIs into structured formats [91]. ML algorithms using supervised and unsupervised learning can process these data to produce a predictive model for content personalization.

User features can be matched to content features (whether video, text, infographics, or audio) to create a model predicting which content is likely to be engaging to which people. Content features need not be limited to the content or design features identified in the *Content Personalization* section. Meta-level information encompassing object identification (colors, shapes, and texture), person or face identification, motion features, patterns, textual analysis, medical tags, higher semantic meaning, and significantly more may be extracted and analyzed. The content for patients can be created with these specific features in mind. Recommender systems could automatically predict other content that is useful and engaging to patients, conveying education that is likely to affect them.

Metrics related to view count, likes, shares, and positive comments have traditionally been used as an indicator of popularity, but they may only provide a superficial measure of engagement and fail to capture key outcomes for patients. Similarly, no single outcome metric is likely to be sufficient. Possible surrogate measures to consider include shared decision-making [27], patient satisfaction [92], objective clinical outcomes and symptoms [93], changes in attitude and behavior [94,95], and physiological signals [96]. These factors can aid in content personalization.

Harnessing data from personal digital devices such as wearables, phones, and computers has led to research into digital phenotyping and personal sensing, which refer to the analysis of data streams from personal devices to build a human phenotype by identifying behaviors, traits, thoughts, and feelings [97,98]. This field has been adopted predominantly in psychiatry, where the objective identification of behavior patterns can aid in the diagnosis and stratification of mental health conditions, as well as their treatment (digital health interventions) [97]. In a recent study of internet-based cognitive behavioral therapy, ML was used to identify different behavior patterns among segments of patients, consisting of low engagers, late engagers, high engagers with rapid disengagement, and the highest engagers [99]. Each patient subtype was more likely to engage with different intervention tools (eg, core modules, goal-based activities, mood trackers, and mindfulness tools), leading to varying improvements in depression and anxiety symptoms. The authors concluded that this information could be used to tailor specific intervention types to different patient subtypes to improve engagement and adherence to treatment.

There are clear similarities between these digital health concepts and the proposed model to personalize web-based health care content. In particular, ML can be used to analyze data streams that include sensor measurements, user activity on personal devices, and user-generated content to identify individual behavior patterns. This can then be used to personalize interventions, of which personalized content could form a part of the intervention, or, at the very least, to inform patients about their health and engage them in making healthy behavior choices.

The successful implementation of big data and ML in personalizing web-based content requires the input and collaboration of several multidisciplinary stakeholders [100]. Health care professionals must produce accurate and engaging user-centered content, which is consumed by patients who can use recommender systems to discover related content and are also able to create content on their own. ML algorithms based on the model described in Figure 1 were designed by computer scientists and ML engineers and further optimized by several data streams provided by patients and health care organizations. There should be ongoing collaborative research between clinicians and computer scientists to take advantage of developments in ML, such as the use of deep learning.

However, current inadequacies in the digital infrastructure of health care systems can pose a significant challenge to this process. For example, as outlined in the UK government policy paper on their future digital strategy plan [101], patient data are often stored in disparate systems between different hospitals and health care settings that are unable to communicate with each other. One of the priorities should, therefore, be to create data standards that facilitate the interoperability of patient health records, which would enable seamless access, storage, and processing at scale. It is promising that government agencies have already taken steps to outline frameworks to achieve secure access, interoperability, and sharing of health-related patient data [101,102].

Other drawbacks of big data and personalized health care must also be considered in addition to the benefits. Maintaining the privacy and security of sensitive patient data is paramount and poses significant challenges with the volume of data recorded from an increasing number of sources. No single legal or ethical framework covers all aspects of health information privacy [103]. Furthermore, many laws are outdated and insufficient for the current era of big data, which includes user-generated data (eg, wearables and sensors) and nonhealth information that can lead to health inferences (eg, social media habits) [104]. Therefore, governments and health care bodies must also act as key stakeholders to ensure that laws are updated to allow ML to be harnessed for the benefit of patients while maintaining privacy and security. This may necessitate the development of oversight agencies to strictly regulate the use of ML, as well as collaboration with cybersecurity experts [100]. The principles of consent in digital data research and use need to be established and will require input from governments, national data regulators, medical ethicists, legal experts, and, most importantly, patients [105].

There are several principles for maintaining private and secure data, including collecting data from trusted sources, encrypting and anonymizing stored data, maintaining strict authorization and access control, and securing processing environments [106]. However, a cybersecurity report in 2016 revealed a 320% year-on-year increase in breaches of protected health information in US hospitals, with 81% of breached records resulting from hacking attacks [107]. This compromised over 16 million individual patient health records, indicating a pressing need to continue monitoring and developing security systems in the face of both malicious and unintentional data breaches.

## Conclusions

The proliferation of web-based content and increased participation of patients in interacting with said content provides an opportunity to understand what features of content are engaging to people. Harnessing ML technologies to process *big data* in health care will allow health care providers and other users to create and contribute to personalized content. These insights may be leveraged to facilitate patient activation and enable patients to make healthy choices, ultimately improving outcomes.

## Abbreviations

API: application programming interface
ML: machine learning
